# Quality Assessment of Shock Videos on Video Sharing Platforms: Cross-Sectional Study

**DOI:** 10.2196/76715

**Published:** 2025-12-30

**Authors:** Wenxin Wang, Luping Cheng, Xia Hu, Chuanliang Pan

**Affiliations:** 1 North Sichuan Medical University, Nanchong, China; 2 Chengdu Third People's Hospital, No. 82 Qinglong Street, Qingyang District, Chengdu, Sichuan Province, 610031, China, 86 18111586553

**Keywords:** quality assessment, shock, video, GQS, mDISCERN, PEMAT A/V, Global Quality Score, Patient Education Materials Evaluation Tool for Audiovisual Content

## Abstract

**Background:**

As a highly lethal circulatory failure syndrome, the pathophysiological mechanisms of shock can lead to multiple organ dysfunction syndrome (MODS), which significantly increases the demand for intensive care and the length of hospitalization. There is therefore an urgent need for the public to be informed about health-related issues. In recent years, videos have become a significant medium for health education, and this study aimed to evaluate shock-related videos on video sharing platforms.

**Objective:**

The objective of this study is to identify the top 100 videos related to impact on TikTok, Bilibili, and Xiaohongshu. These videos will then be assessed in terms of their effectiveness and credibility. Following this evaluation, relevant recommendations will be provided.

**Methods:**

The study included a search for videos related to shock on the three video-sharing platforms: TikTok, Bilibili, and Xiaohongshu. The Global Quality Score (GQS) and mDISCERN tools were used to evaluate the credibility and quality of the videos, in addition to employing the Patient Education Materials Evaluation Tool for Audiovisual Content (PEMAT-A/V). Finally, the video was evaluated by examining disease definitions, clinical manifestations, risk factors, assessment, management, and outcomes.

**Results:**

A total of 244 videos (TikTok:87, Bilibili:80, Xiaohongshu:77）were retrieved from the three platforms. The overall video quality was found to be moderately low. The majority of videos were uploaded by health advocates (n=102, 41.8%) and health professionals (n=98, 40.1%). The individual video sources of the GQS were of lower quality (1-3), the mDISCERN scores were moderate (2-4), and the quality of individual users is higher than that of organizational users. The PEMAT A/V scores were as follows: in the overall comprehensibility evaluation, 91% (220) videos of the scores were above 70%; in the actionability evaluation, 65% (157) videos of the scores were below 70%. It should be noted that the actionability scores for different video sources were generally low. In 172 videos (70.4%), the definition of shock and its clinical manifestations were explained in detail, while in 137 videos (56.1%), the definition of shock and its clinical manifestations were also clearly explained. The majority of videos provided a relatively comprehensive explanation of the definition of shock and its clinical signs and symptoms.

**Conclusions:**

Our study have demonstrated that the content and information quality of shock videos is unsatisfactory, as a general rule. This underscores the necessity for pertinent regulatory bodies to oversee the caliber of health-related videos, and for content creators to enhance the quality of their content.

## Introduction

### Background

Shock is defined as a systemic hypoxic crisis triggered by a breakdown of circulatory system function. The central paradox is a serious imbalance between tissue oxygen demand and blood oxygen supply capacity. If this is not corrected in time, irreversible organ damage will ensue. The underlying causes of shock are multifactorial in nature, encompassing a wide spectrum of etiologies, including hypovolemic, cardiogenic, obstructive, and distributive causes, which may manifest in isolation or in conjunction [1]. Irrespective of the nature of the shock, its consequences are invariably fatal. For instance, in cases of cardiogenic shock, the heart’s capacity to pump blood is significantly compromised, resulting in systemic organ dysfunction. The mortality rate remains high, estimated at approximately 50% [2]. Research indicates that key elements of optimized treatment for cardiogenic shock include early assessment and timely initiation of acute mechanical circulatory support devices [3], and early diagnosis and treatment of pediatric sepsis and infectious shock can improve patient outcomes [4]. Therefore, early recognition and rapid response are critical for improving patient outcomes. The timely recognition of shock and subsequent prompt emergency treatment (including on-site resuscitation, rapid hospitalization, and other related measures) is contingent upon public health awareness. It is imperative to emphasize that enhancing the public’s awareness of shock-related health issues is of great significance.

In recent years, videos have attracted mounting interest as a medium for health information dissemination. Empirical evidence has demonstrated the efficacy of brief audiovisual presentations in enhancing viewers’ health knowledge and awareness, with a particular emphasis on the popularization of first aid knowledge [5]. The rapid dissemination and high acceptance of videos renders them a significant source of health information, particularly in the context of disseminating knowledge about complex diseases such as shock [6]. The dissemination of such information has the potential to enhance public awareness of diseases, facilitate the timely treatment of patients, and improve patient outcomes. Such studies can provide a valuable reference point for enhancing public awareness of shock and offer data-driven support for the development of suitable health education strategies [5,7]. Nevertheless, it is imperative to direct particular attention to the quality of the information presented in these videos. The increasing prevalence of video content has the capacity to disseminate health-related health information, which may consequently mislead patients in their disease management [8,9]. While videos may facilitate timely access to medical information for the general population, they may also result in delays to treatment. Consequently, it is imperative to evaluate the quality of shock-related videos. However, to the best of the present author’s knowledge, there are currently no studies evaluating the quality of shock-related short videos. The objective of this study is to assess the health information in videos related to shock, provide data support for existing health information, and formulate relevant recommendations.

The approach adopted in this study is advantageous in that it facilitates the rapid collection of a substantial amount of data, enables efficient statistical analysis, and ensures the reliability and validity of the results [10]. The present study will analyze the content of videos with a view to revealing their role and existing issues in the dissemination of health information related to shock. The study will provide a scientific basis for the effective use of short videos in public health education. Key issues that will be examined include the accuracy of information in short videos, their professionalism, and their influence on viewers’ behavior [11,12]. Finally, the relevant results will be analyzed, and appropriate recommendations will be proposed.

### Objective

TikTok, Bilibili, and Xiaohongshu have demonstrated significant efficacy in disseminating educational video content in prior studies [13,14], which informed our selection of these platforms for the current research. The objective of this study is to identify and collect the top 100 videos related to shock on the TikTok, Bilibili, and Xiaohongshu platforms. These videos will then be assessed in terms of their effectiveness and credibility, and recommendations will be provided based on the findings.

## Methods

### Ethical Considerations

This project’s research content is publicly disseminated video content on public network platforms, and it does not involve the collection or use of any personal privacy information. The research process did not involve any intervention, investigation or potential risk to individuals, nor did it involve the use of data relating to human subjects or patients, so it did not affect any individuals. Consequently, this study is not subject to ethical review.

### Collection of Data

In order to guarantee the objectivity of data collection, the research team strictly implemented the following standardized preprocessing procedures prior to implementing the retrieval operation. In order to rectify the issue, the following three steps must be taken: (1) the factory settings of all mobile terminals must be restored; (2) the browsing history and cache data of the devices must be completely cleared; and (3) all the platform accounts must be logged out of. In order to circumvent the possibility of potential impact on the results, the study proceeded to retrieve the top 100 videos from TikTok, Bilibili, and Xiaohongshu by conducting a search for the Chinese keyword “休克.” TikTok and Xiaohongshu have opted for comprehensive sorting, while Bilibili has opted for default sorting. The entire search was conducted over the course of seven days, from 5th March to 11th March 2025. The videos were then categorized by three qualified doctors with extensive experience working in tertiary care hospitals in the Department of Critical Care Medicine. The integrity of the video is based on classification, with preliminary decisions made by three clinicians. Any discrepancies are resolved through consensus among the three clinicians. The consistency scores (Cohen κ) for GQS, mDISCERN, PEMAT-U, and PEMAT-A, completed by two doctors, were 0.766, 0.627, 0.839, and 0.802, respectively. These results indicate that GQS and mDISCERN have strong consistency, while PEMAT-U and PEMAT-A have very strong consistency. The following attributes are contained in each video data set: publisher’s profile (comprising account nicknames, personal profiles and official authentication logos); the content release time, the current fan base, the video duration information, and the user interaction metrics (including the number of likes, favorites, share spreads and comments). The data were recorded using Microsoft Excel.

### Inclusion and Exclusion Criteria

Videos that provide knowledge about shock-related topics. The exclusion criteria were as follows: (1) videos with repetitive content; (2) videos containing advertisements or commercials; and (3) videos unrelated to the topic.

### Assessment Methodology and Process

The video publishing body is divided into two major categories : individual users and institutional accounts. In accordance with the findings of preceding studies by Cuiet al [15] and Liu et al [16], the following professional evaluation system is used for multidimensional quality testing. The credibility of the videos was evaluated using the modified DISCERN criteria. The information quality of the videos was evaluated using the Global Quality Score (GQS) criteria. The videos were evaluated using the Patient Education Materials Evaluation Tool for Audiovisual Content (PEMAT-A/V). The mDISCERN tool was originally developed for the purpose of evaluating the quality of written health information concerning treatment regimens [17]. The mDISCERN tool from DISCERN was used to analyze the credibility and quality of the video. The efficacy of this approach has been demonstrated in the evaluation of health-related video material on diverse platforms, including *Helicobacter pylori*, tachycardia, thyroid cancer, and other disorders in assessing video use [18-20]. The mDISCERN tool is composed of five inquiries: (1) Are the objectives clear and realized?; (2) Were reliable sources of information used?; (3) Was the information presented balanced and fair?; (4) Are alternative sources of information listed? Are other sources of information listed for patient reference? (5) Are areas of uncertainty mentioned? The GQS is another widely used tool for assessing the quality of health information in videos, with response selection based on a 5-point scale from 1 (poor quality) to 5 (good quality) [21,22]. The PEMAT-A/V [23] was used to evaluate the comprehensibility and actionability of the video material. The PEMAT-A/V comprises 17 questions; 13 assess the patient’s comprehension of the health information and 4 evaluate the actionability of the recommendations in the video.

Finally, the video was evaluated by examining disease definitions, clinical signs and symptoms, risk factors, assessment, management, and outcomes. The allocation of scores was categorized into three primary sections: no content (0 points), content (1 point), and more content (2 points). Consequently, the content, reliability, and quality of the videos were assessed by two clinicians based on the mDISCERN, GQS scoring systems, and PEMAT-A/V. Continuous variables are expressed as mean and SD or median and IQR. Categorical variables were expressed as numbers with corresponding percentages. Prior to the evaluation, the two clinicians familiarized themselves with the formal descriptions of the utilization tools and discussed the use of the tools to assess the content and quality of the videos.

### Data Analysis

Data were analyzed using SPSS (version 30.0; IBM Corp) for statistical correlation and analysis. Data are presented as numbers (percentage), mean (SD), or median (IQR). Kolmogorov-Smirnov test (n>50) or Shapiro-Wilk test (n≤50) were used to assess whether the data followed a normal distribution. The Mann-Whitney *U* test was used to compare two groups of data. The Kruskal-Wallis H test is used to compare three or more groups of data, with Dunn test subsequently used as post-hoc test for pairwise comparisons. The interrater reliability of the two evaluators’ scores was assessed using Cohen χ test.

## Results

**Figure 1.**
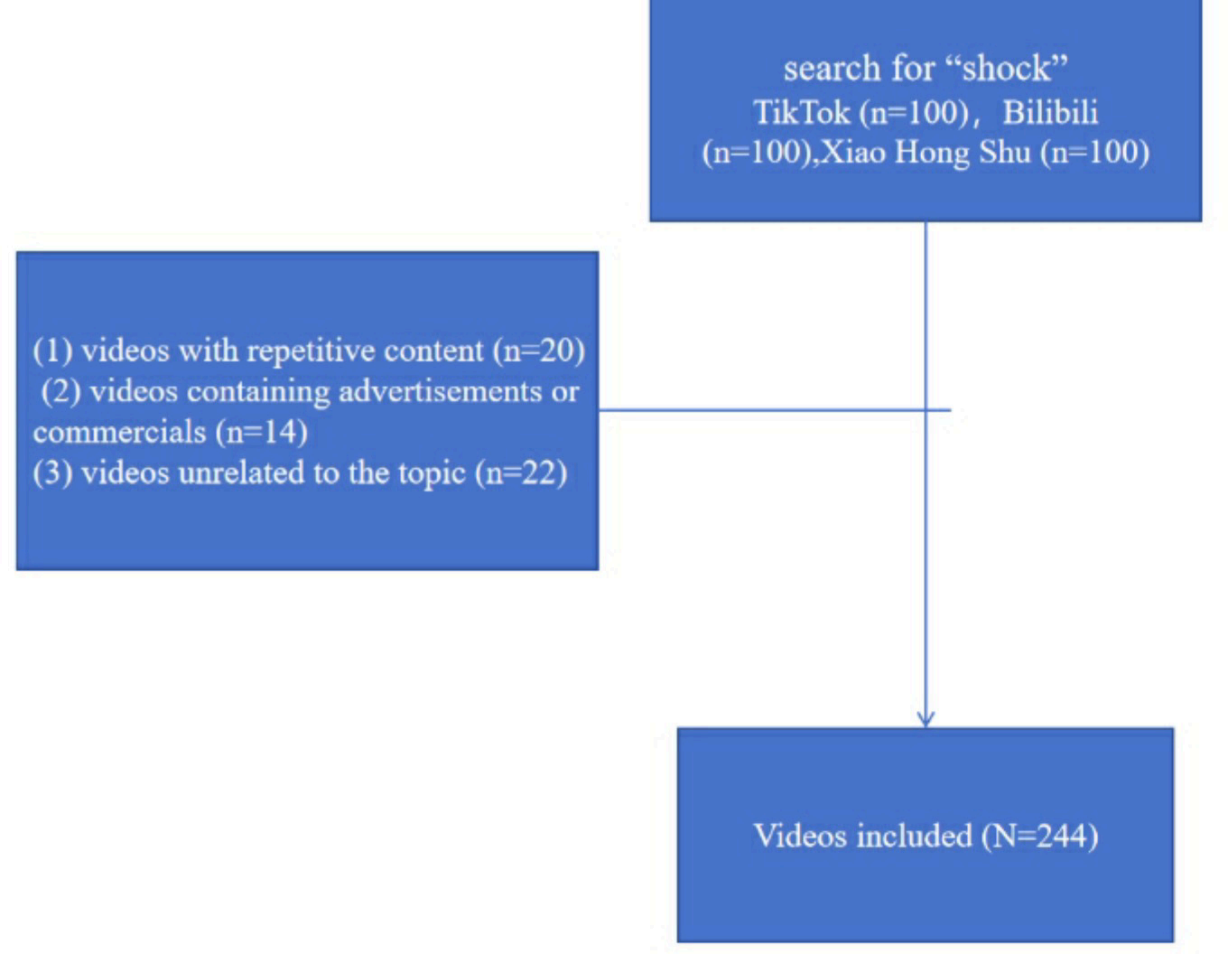
Patient flowchart.

A total of 244 videos (TikTok:87, Bilibili:80, Xiaohongshu:77）were retrieved from the three platforms and included in the subsequent data analysis (Figure 1). The categorization of video sources according to account information from the three platforms revealed two categories: individual users and institutional accounts. A further categorization of individual users is possible, distinguishing between health professionals, health advocates, and patients. A similar classification system was applied to institutional accounts, which were divided into three categories: news organizations, nonprofit organizations, and for-profit organizations. The results of the video analysis (Table 1) demonstrated that health professionals were the most prolific contributors, uploading a total of 102 videos (41.8%).

In the runner-up position were health advocates a total of 98 videos (40.1%), and patients, who uploaded a total of 12 videos (4.9%). Amongst institutional accounts, nonprofit organizations uploaded the most videos related to shock, with a total of 18 videos (7.4%). Subsequent to this, for-profit organizations uploaded 10 videos (4.1%) , while news organizations uploaded 4 videos (1.7%).

Search strategy for videos on shock. TikTok excluded 5 videos with repetitive content, 3 videos containing advertisements or commercials, and 5 videos unrelated to the topic. Bilibili excluded 6 videos with repetitive content, 4 videos containing advertisements or commercials, and 10 videos unrelated to the topic. Xiaohongshu excluded 9 videos with repetitive content, 7 videos containing advertisements or commercials, and 7 videos unrelated to the topic.

**Table 1.** Descriptions of video sources.^a^

Source type	Description	Video, n (%)
Health professionals	Individuals who have received specialized education in healthcare (eg, doctors, nurses)	41.8 (102)
Heath advocate	Transform knowledge that one understands personally into knowledge that can be comprehended by the general public.	40.1 (98)
Patient	Have suffered from the corresponding disease before	4.9 (12)
Nonprofit organizations	Organizations that exist to serve a specific charitable, educational purpose (eg, hospitals, colleges)	7.4 (18)
For-profit organizations	Companies that operate to generate income	4.1 (10)
News organizations	Organizations whose main purpose is to gather and distribute news and information (TV account, newspaper)	1.7 (4)

a The following section provides a detailed explanation of each video source category, along with their respective percentage shares.

As demonstrated in Table 2, the duration of videos uploaded on video sharing platforms varied significantly across the sample. The median length of upload dates for health advocates was the longest at 562 days, followed by news organizations at 501 days, and the shortest for non-profit organizations at 146 days. The median duration of videos was the longest for patients at 146 seconds, followed by non-profit organizations at 103.5 seconds, and the shortest for health advocates at 40.5 seconds. The most prevalent engagement patterns were observed among news organizations, with ’liking’, 'favouriting’ and commenting. In contrast,, for-profit organizations demonstrated the least engagement, with the least popular patterns being ’liking’, 'favouriting’ and commenting. Health professionals had the highest number of followers, with a median value of 6654. Subsequent to this, news organizations followed, patients and health advocates, with 2173, 2158, and 2060 followers, respectively.

**Table 2.** Video characteristics.

Source type	Account fans	Length	Video likes	Collections	Video comment	Days on platform
Health professionals, median (IQR)	6654（613 to 135200）	76（7 to −3648）	110（0 to 8700）	30（0 to 31280）	6（1 to 3497）	299（8 to 1650）
Heath advocate, median (IQR)	2060（219 to 9658）	40.5（8 to 7020）	35（0 to 414000）	61（0 to 15000）	2（0 to 10000）	562（1 to 8654）
Patient, median (IQR)	2158（180 to 8983）	146（12 to 255）	29（1 to 4706）	6（1 to 645）	5（0 to 1341）	170（1 to 770）
Nonprofit organizations, median (IQR)	1624（490 to 16750）	65.5（10 to −3628）	55（0 to 3648）	47（0 to 2089）	1.5（0 to 41）	199（46 to 1577）
For-profit organizations, median (IQR)	700（190 to 7207）	103.5（8 to 356.5）	10（2 to 52）	7（0 to 106）	0（0 to 10）	146（3 to 947）
News organizations, median (IQR)	2173（341 to 12,405,804）	97（7 to 1500）	4069（0 to 38,000）	533（3 to 2364）	180（44 to 2535）	501（329 to −1430）

Differences between groups were analyzed based on GQS and mDISCERN scores. Figures A and D were analyzed using the Mann-Whitney U test. Figures B, C, E, and F were analyzed using the Kruskal-Wallis H test, followed by Dunn’s test subsequently used for post-hoc comparisons with *P*<.05.

**Figure 2.**
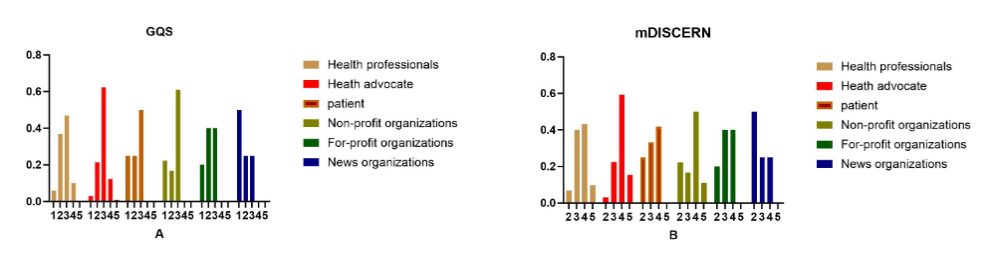
Frequency distribution of GQS and mDISCERN scores. GQS: Global Quality Score.

**Figure 3.**
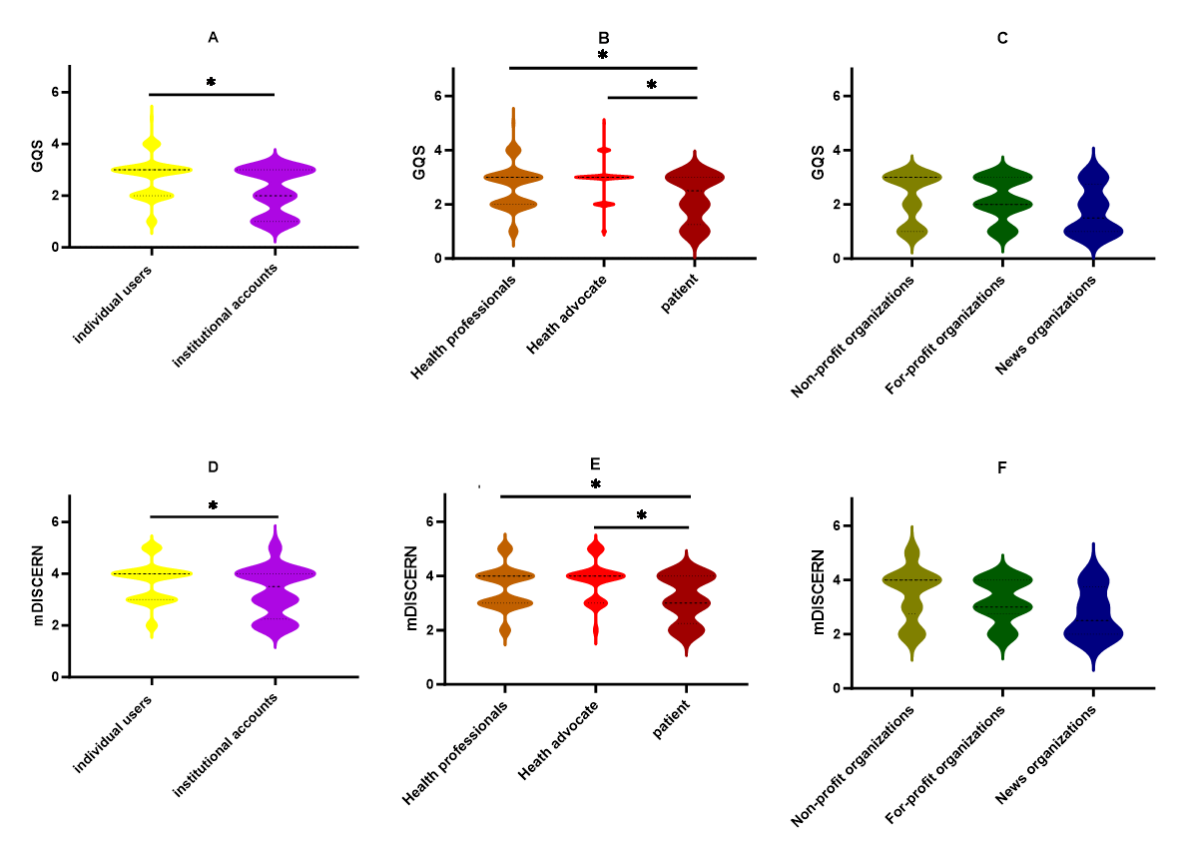
Comparison between mDISCERN scores and global quality scores (GQS) across video sources.

As illustrated in Table 3 and Figure 2, the GQS scores for all video sources are relatively low, with health advocates demonstrating the highest quality, health professionals the second-highest, and news organizations the lowest. The mDISCERN scores were moderate; with health advocates exhibiting the highest credibility; health professionals, the second-highest; and news organizations the lowest. Moreover, the mDISCERN frequency distribution indicates that patients, news organizations, nonprofit organizations, and for-profit organizations have low credibility. Figure 3 compares the reliability and quality of information from different video sources. A subsequent comparison of individual users with organizational users revealed that both GQS and mDISCERN scores were higher for individual users than for organizational users (*P*<.05). Within the individual user category, health advocates and health professionals demonstrated higher information quality, while patients exhibited lower information quality (*P*<.05). However, no statistically significant differences were observed within the organizational category. The overall quality level of mDISCERN is moderate, while the quality level of news organizations is lower. Within the individual user category, health advocates and health professionals were found to have higher credibility than patients (*P*<.05), while no statistically significant differences were observed within the organizational user category.

**Table 3.** GQS^a^ and mDISCERN scores. Mean (SD) and median (IQR) values of GQS and mDISCERN scores based on video classification.

Source type	GQS		mDISCERN scores	
Mean (SD)	Median (IQR）	Mean (SD)	Median (IQR)
Health professionals	2.65 (0.79)	3 (2‐3)	3.56 (0.76)	4 (3‐4)
Heath advocates	2.87 (0.70)	3 (2.75‐3)	3.87 (0.70)	4 (3‐4)
Patients	2.25 (0.87)	2.5 (1.25‐3)	3.17 (0.84)	3 (2.25‐4)
Non-profit organizations	2.44 (0.92)	3 (1.75‐3)	3.5 (0.99)	4 (2.75‐4)
For-profit organizations	2.2 (0.79)	2 (1.75‐3)	3.2 (0.79)	3 (2.75‐4)
News organizations	1.75 (0.96)	1.5 (1‐2.75)	2.75 (0.96)	2.5 (2‐3.75)

a GQS: Global Quality Score.

**Figure 4.**
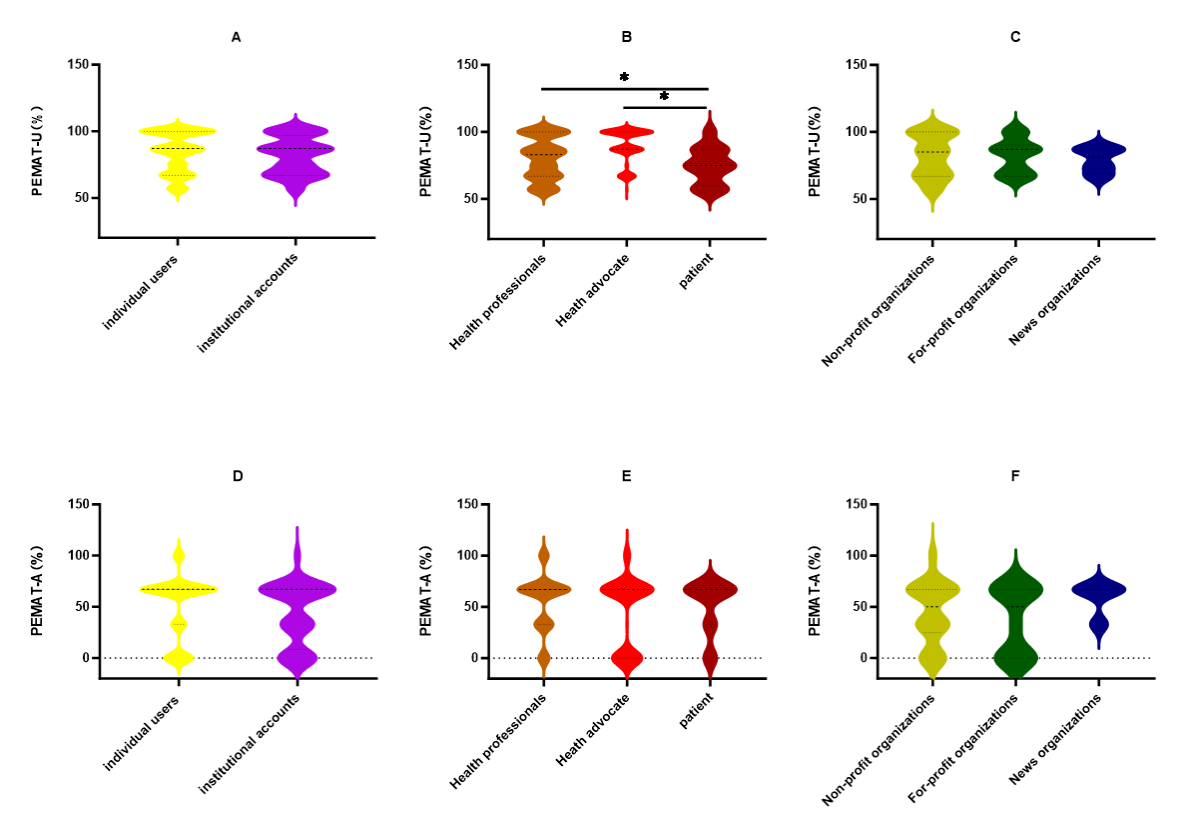
Comparison between PEMAT-U scores and PEMAT-A scores (GQS) across video sources. GQS: Global Quality Score; PEMAT-A: Patient Education Materials Assessment Tool for Audio Visual Materials; PEMAT-U: Patient Education Materials Assessment Tool-Understandability.

As demonstrated in Table 4, a considerable proportion of the PEMATA/V scores surpassed the overall understandability threshold of 70%, with 91% (n=220) videos of the scores falling into this category. Furthermore, 65% (n=159) videos of the scores did not exceed 70% in terms of actionability, while 73% (n=178) videos of the scores exceeded 70% at the overall level. As demonstrated in Figure 4, there is no statistically significant difference between individuals and organizations in terms of understandability scores. However, among individual users, health professionals and health advocates demonstrated the highest levels of understanding, in comparison to patients (*P*<.05). The study revealed no statistically significant differences in actionability scores between individuals and organizations, nor within each group.

**Table 4.** PEMAT scores presented in the video.

Scale, score	Rate n (%)
PEMAT-U^a^	
0‐60	22 (9)
61‐80	66 (27)
81‐100	156 (64)
PEMAT-A^b^	
0‐50	86 (35)
67‐75	141 (58)
100	17 (7)
PEMAT-T^c^	
0‐60	42 (17)
61‐80	156 (64)
81‐100	46 (19)

ab PEMAT-U: Patient Education Materials Assessment Tool-Understandability

b PEMAT-A: Patient Education Materials Assessment Tool for Audio Visual Materials.

c PEMAT-T: Patient Education Materials Assessment Tool for Telehealth.

Differences between groups were analyzed based on PEMAT-U scores and PEMAT-A Scores. (Figures 4A and 4D) were analyzed using the Mann-Whitney U test. Figures (Figure 4B, 4C, 4E, 4F) were analyzed using the Kruskal-Wallis H test, followed by Dunn’s test subsequently used for post-hoc comparisons (*P*<.05).

The frequency of myopic shock, as outlined in Table 5, encompasses six fundamental domains: disease definition, clinical manifestations, risk factors, assessment, management, and outcome. The results demonstrated that the definition of shock and its clinical manifestation were clearly explained in 172 videos (70.4%) and 137 videos (56.1%), respectively, while a few videos (26%, 10.7% and 12, 4.9%) had no content. The analysis revealed that approximately half of the videos contained content on risk factors (n=146, 59.8%), management (n=122, 50%), and assessment (n=142, 58.1%). In contrast, approximately half of the videos (n=124, 50.8%) did not include content on outcomes.

**Table 5.** Completeness of video content. The six aspects of the video content are indicated by percentages.

Content	Definition, N (%)	Clinical manifestation, n (%)	Risk factors, n (%)	Evaluation, n (%)	Management, n (%)	Outcomes, n (%)
No content	26 (10.7）	12 (4.9）	86 (35.2）	95 (38.9）	112 (45.9）	124 (50.8）
Some content	46(18.9）	95 (38.9）	146 (59.8）	142 (58.1）	122 (50.0）	109 (42.2）
Extensive content	172 (70.4）	137 (56.1）	12 (4.9）	7 (2.9）	10 (4.1）	11 (4.5）

## Discussion

### Principal Findings

The physiological impact of shock on patients is extremely severe, leading to multiple organ failure, and the economic burden, including hospitalization and long-term rehabilitation costs, significantly increases the strain on the healthcare system [24]. Despite the advances in the diagnosis and management of shock, including pharmacological treatment, fluid resuscitation, and surgical intervention, there are still deficiencies in the early recognition and rapid response, resulting in a poor prognosis for patients [25]. Research also indicates that early diagnosis can improve patient outcomes [7,8]. Early diagnosis and rapid treatment are critical, but timely identification of shock and swift implementation of measures depend on public health awareness, with short videos playing a key role as an important source of health information [8,9]. Consequently, the study of health information dissemination for shock, particularly the use of short videos, is of particular importance in enhancing public awareness of shock [26]. This study used an observational review design with the aim of examining the current status of videos as a shock health information resource and assessing their effectiveness and potential impact in public health education. The content and quality of videos were analyzed to understand the role and problems of videos in health information dissemination.

The current user scale of video applications shows a rapid growth trend. This emerging mode of communication in the field of health knowledge popularization shows a unique advantage: video will transform complex medical concepts into dynamic images, three-dimensional demonstrations and scenario-based explanations. The convenience for users to obtain health information has been significantly improved. While this medium has undoubtedly facilitated the dissemination of health-related knowledge, it also increases the risk of the public obtaining incorrect health information [8,9]. To date, no studies have evaluated videos related to shock, the necessity for rigorous evaluation through relevant studies is therefore paramount. The impact of shock-related videos on social media platforms such as TikTok, BiliBili, and Xiaohongshu remains to be elucidated. This study comprehensively assessed 244 shock videos on TikTok, BiliBili, and Xiaohongshu, using rigorous content and quality evaluation methodologies.

The findings of the present study demonstrated that these 244 videos amassed a total of 129,664,761 followers, 879,855 likes, 127,345 collections, and 35,226 comments. Video sharing platform, represented by TikTok (Shake), Bilibili and Xiaohongshu, have transformed professional content into visualizations and become an important channel for accessing information. The results of the study indicate that health professionals and health advocates dominate the content, a phenomenon that differs from previous studies [11,27], some videos uploaded from patients, news media, nonprofits, and for-profit organizations, suggesting that people are willing to share their health knowledge on the Internet. Despite the fact that health professionals boast a greater number of followers than news organizations, news outlets receive a greater number of likes, saves and comments, and are more disseminative. This finding is consistent with the conclusions of Cui et al [15] and Jun Qiu et al [27]. It is therefore vital that health professionals enhance their ability to promote health knowledge. However, the study’s findings reveal that the median upload duration for the majority of videos exceeded 100 days, with some sources displaying an upload duration of over 500 days. This suggests a tendency for videos pertaining to shock to undergo less frequent updates. The dissemination of video recordings that have been in circulation for an extended period may result in the dissemination of outdated knowledge, thereby necessitating the prompt updating of such content by the public. The platform has the capacity to provide relevant incentives to encourage individuals to disseminate health knowledge related to shock.

According to previous studies by Cui et al and Liu et al [15,16], we selected mDISCERN, GQS, and PEMAT-A/V to evaluate video quality. The Mann-Whitney U test is used to compare two sets of data, while the Kruskal-Wallis test is used to compare three or more sets of data. The Dunnet multiple comparison test is used for two-way comparisons between two groups.

Initially, the credibility of the video content was assessed using mDISCERN. The majority of video sources exhibited mDISCERN scores that approximated the medium quality threshold. The credibility of individual users was found to exceed that of organizational accounts. Additionally, within the cohort of individual users, health professionals and health advocates were found to possess greater credibility than patients. This finding is consistent with the observations reported by Lai Y [19]. It is recommended that the platform enhance its content review process for patient and organizational users. This is attributable to the capacity for non-professionals to disseminate misinformation, which may occasion delays in treatment.

The GQS validation tool was utilized to assess the quality of the videos. It was found that individual users exhibited higher quality than organizational accounts. Individual users have been found to demonstrate higher quality than organizational accounts. Furthermore, among individual users, health professionals and health advocates have been found to demonstrate higher quality than patients. However, the mean score for the majority of videos is 3 or below, indicating that video quality is at a moderately low level, consistent with the findings of Collà Ruvolo et al [28]. This finding suggests a need for further development and refinement of trauma-related videos to enhance their effectiveness. Based on our analysis of the videos, we have identified two potential issues: (1) health professionals and health advocates use technical terms that are difficult for the general public to understand and (2) Patients and individuals lacking professional knowledge may produce videos containing inaccurate information. Therefore, health professionals and health advocates must share their knowledge in a way that is easier for the general public to understand and access. Additionally, relevant platforms must conduct more rigorous content reviews to ensure the public can access accurate information.

In conclusion, the third instrument utilized was PEMAT-A/V, which was used to evaluate the educational pertinence of the videos. As posited by Shoemaker et al [23], the definition of video content as both understandable and actionable was proposed. Furthermore, it was indicated that a PEMAT score in excess of 70% would be indicative of a professional video. This finding demonstrated that shock-related videos were comprehensible but not directly applicable. The two groups of " health professionals” and “health advocates” demonstrated a significantly higher level of understanding of the information compared to patients, consistent with previous research [18]. The study’s findings suggest that the comprehensibility of the information meets established standards; however, the actionable content is found to be deficient. As demonstrated by Kanner et al [29], actionable content is relatively restricted. Morra et al [30] also noted that the study topic may lead to differences in related scores. The relatively low GQS scores mentioned above may also be related to the actionable content of the reviewed videos. It is evident that a significant proportion of videos offer merely a cursory introduction to the subject, with a paucity of actionable content. This is exemplified by the absence of emergency procedures, such as lying down, elevating the legs, maintaining body temperature, monitoring breathing, and performing cardiopulmonary resuscitation when necessary. It is imperative to enhance the practicality of these systems, a necessity that is especially pronounced in trauma-related scenarios where immediate attention is paramount. The practicality of video content from various sources should be enhanced, for example by incorporating demonstrations by health professionals and health advocates.

The study results indicate that the majority of videos (172, accounting for 70.4%; 137, accounting for 56.1%) provided clear explanations of the definition of shock and its clinical manifestations, covering all aspects of the disease, which is consistent with un Qiu et al [27]. The dissemination of knowledge regarding the mechanisms of shock is predominantly facilitated by the diversification of science communication channels, with online media assuming a particularly prominent role in this regard. Nevertheless, the dissemination of knowledge pertaining to risk factors, management, and outcomes continues to necessitate ongoing enhancement. Platforms have the capacity to engage in collaborative endeavors with pertinent professionals, thereby facilitating the dissemination of such knowledge.

The present study is subject to several limitations. First, it is important to note that this is a cross-sectional study, which is limited in its ability to reflect results at a single point in time. This inherent limitation makes it difficult to replicate and unable to conduct dynamic analysis modeling of user interaction behavior. Furthermore, it cannot perform causal analysis. Second, there is a possibility of bias in the classification of videos and diseases across the six dimensions examined in this study. Third, the present study systematically examined the quality of content themed around ’shock’ on video sharing platform such as TikTok, Bilibili, and Xiaohongshu. However, the review mechanisms of these platforms differ, which may have influenced the study results. Fourth, due to the limitations of the platform itself, it is impossible to obtain accurate viewing figures for videos, which may result in the videos included not being sufficiently disseminated, thereby affecting the results. Fifth, owing to defects inherent in the platform, a significant proportion of user identities are incomplete, thus impeding the execution of subgroup analyses, including those pertaining to the classification of doctors.

### Conclusion

The present study analyzed a total of 244 videos (TikTok:87, Bilibili:80, and Xiaohongshu:77）related to shock, obtained from the three video sharing platforms. The analysis indicated that the content and information quality of the videos was, overall, not satisfactory. The quality of the video was found to be contingent upon the source of the videos in question. With regard to the reliability and quality of the videos, the study found that overall, these were moderately low, although videos from health advocates and health professionals ranked first and second, respectively. This highlights the necessity for the first relevant regulatory authority to oversee the quality of health-related short videos, thereby assisting the public in achieving early prevention and treatment of diseases. The majority of videos fail to incorporate actionable elements, and it is imperative that they are improved in terms of their presentation of content in order to enhance their actionability. In addition, health professionals and health advocates should intensify their efforts in dissemination and enhance the actionability of the content.

## Abbreviations

GQS: Global Quality Score
MODS: multiple organ dysfunction syndrome
PEMAT-A/V: Patient Education Materials Evaluation Tool for Audiovisual Content
PEMAT-U: Patient Education Materials Assessment Tool-Understandability
